# Labour politics and the EU’s new economic governance regime (European Unions): a new European Research Council project

**DOI:** 10.1177/1024258918770046

**Published:** 2018-05-10

**Authors:** Roland Erne

**Affiliations:** UCD School of Business and Geary Institute for Public Policy, Belfield, Dublin, Ireland Email: Roland.Erne@ucd.ie

European trade unions play a major role in democratic interest intermediation. This role is currently threatened by the increasingly authoritarian strain in the EU’s new economic governance (NEG). This research project aims to explore the challenges and possibilities that the NEG poses to labour politics. Until recently, European labour politics has mainly been shaped by horizontal market integration through the free movement of goods, capital, services and people. After the financial crisis, the latter has been complemented by vertical integration effected through the direct surveillance of member states. The resulting NEG opens contradictory possibilities for labour movements in Europe.

On the one hand, the reliance of the NEG on vertical surveillance makes decisions taken in its name more tangible, offering concrete targets for contentious transnational collective action. On the other hand, however, the NEG mimics the governance structures of multinational firms, by using key performance indicators that put countries in competition with one another. This constitutes a deterrent to transnational collective action. The NEG’s interventionist and competitive strains also pose the threat of nationalist counter-movements, thus making European collective action ever more vital for the future of EU integration and democracy.

This project has the following objectives:

1. To understand the interrelation between the EU’s new ‘vertical’ and existing ‘horizontal’ economic governance and shifts in labour politics triggered by the EU’s NEG;
2. To open up novel analytical approaches that are able to capture both national and transnational social processes at work;
3. To analyse the responses of established unions and new social movements to NEG in different areas of labour politics, economic sectors and governance levels, and their feedback effects on NEG;
4. To develop a new scientific paradigm capable of accounting for the interplay between EU economic governance, labour politics and EU democracy.

## Urgent challenges

This project focuses on the way in which established European trade unions and new social movements respond to the EU’s new economic governance regime. Until very recently, European labour politics has been shaped mainly by EU ‘horizontal’ market integration through the free movement of goods, capital, services and people. Since the Euro crisis however, the latter has been complemented by ‘vertical’ hierarchical integration effected through the direct surveillance of member states’ macroeconomic policies, including industrial relations and social policy. The resulting new EU economic governance regime (NEG) opens contradictory possibilities for labour movements and politics in Europe.

On the one hand, the NEG’s reliance on vertical surveillance makes decisions taken in its name more tangible, thereby offering concrete targets for contentious transnational collective action (Erne, 2008; Erne et al. 2015; Kay, 2015). On the other hand, the NEG mimics the governance structures of multinational corporations (Erne, 2015). By using performance indicators and coercive comparisons that put countries in competition with one another, it therefore implicitly constitutes a deterrent to transnational collective action. Moreover, the interventionist strains and competitive pressures associated with NEG increase the threat of nationalist counter-movements. This is undermining the structuring of the political space along transnational cleavages, namely, the class cleavage. However, the existence of transnational cleavages is a necessary requirement for transnational democracy. As stated by Caramani (2015: 3) two central democratic functions, responsiveness and accountability, ‘are in fact diminished if voters are divided territorially along segmented electorates’.

The labour movement and labour politics are integral to European politics and society. Labour mobilisations that followed the industrial revolution homogenised political attitudes and behaviour within and across countries (Bartolini, 2000; Caramani, 2015, 2004). Furthermore, neither national democratisation processes nor the mid-twentieth-century class compromise, on which Europe’s social models were built, would be conceivable without the social mobilisations of European workers and their organisations at workplaces and in national political arenas (Crouch, 1999). A similar analogy can be made in a transnational context (Erne, 2008). However, labour movements’ capacity to

- structure the transnational political space along class cleavages;
- play a key role in public (and private) interest intermediation;
- enforce class compromises in industrial relations and social policy;

has been seriously challenged.

## Research questions and objectives

These three dimensions of labour politics are currently threatened by a new ‘silent revolution’ (Barroso, cited in ANSA, 2010) in European economic governance. This project therefore aims to explore the tensions, challenges and possibilities that the interventionist turn in the EU’s NEG poses to labour politics in Europe. In the context of increased social and political tensions that are dividing Europeans (Schmidt, 2015; Streeck, 2013, 2015), this project aims to answer the following interrelated **research questions**:

1. Is NEG restructuring the European political space along national or class lines?
2. Are established trade unions and new social movements politicising NEG along national or class lines?
3. What are the consequences of these developments for democracy in Europe?

These questions also address urgent conceptual issues in times when even proponents of neo-functionalist European integration theory envisage the following scenario: ‘first, the collapse of the euro; then of the EU, and, finally, of democracy in its member states’ (Schmitter, 2012: 41).

Even before the Euro crisis and the ensuing silent revolution in European governance, it was argued that the formation of a new European political centre with strong regulatory and judicial capacities would be very problematic. This is because of the deficient parallel ‘system building’ in the field of transnational social integration and democratic participation rights (Bartolini, 2005). Yet, it is conceivable that transnational social integration and democratic participation will emerge after the creation of political authority at the EU level. Whether one is conceptualising *the political* in deliberative-democratic or in power-struggle-oriented terms, one should acknowledge that political authority over a population did not include democratic and social rights from the outset. The formation of political authority has usually been a product of ‘coercion and capital’ (Tilly, 2000). Democratic and social rights followed afterwards as a result of social and political learning processes or struggles by ‘countervailing powers’ (Galbraith, 1952) in response to social tensions created by the making of integrated markets and political authority (Erne, 2008: 18; Habermas, 1996: 506; Marshall, 1992 [1950]).

The formation of much more robust European governance institutions through NEG can also be seen as a precondition for the creation of a transnational democracy. ‘Democracy requires not only a people (*demos*) but also binding rules (*kratos*)’ (Erne, 2008: 18). As democracy is dependent on political authority to enforce the results of democratic consultations, there is a dialectical relationship between popular mobilisations and the creation of political authority, even if few of the participants were really trying to create democratic institutions (Tilly, 2004). Therefore, this project will explore whether labour movements are capable of politicising NEG, which means transforming NEG into a matter of ‘public choice’ (Hay, 2007: 79), through transnational collective action. After all, transnational democracy will not result from theorising alone (Erne, 2008: 18).

This project has therefore the following **objectives**:

1. To understand the interrelation between NEG and existing ‘horizontal’ EU economic governance and the shifts in labour politics triggered by NEG;
2. To open up novel analytical approaches that are able to capture both national and transnational social processes at work;
3. To analyse the responses of established unions and new social movements to NEG in different areas of labour politics, economic sectors and governance levels, and their feedback effects on NEG;
4. To develop a new scientific paradigm capable of accounting for the interplay between EU economic governance, labour politics and EU democracy.

## Conceptual reflections

Politicisation processes and the restructuring of the socioeconomic and political space can be observed at three analytical levels, namely, at individual (micro), organisational (meso) and systemic (macro) level. Most studies in the field have favoured analyses located at either the micro or the macro level (Zürn, 2016). It is quite easy to analyse datasets about changing voter attitudes or to measure the salience of EU-related political issues in media debates (Zürn, 2016). Likewise, the growing socioeconomic polarisation has been well documented and analysed (Galbraith, 2012; Piketty, 2013). Yet, new political and economic polarisations and the emergence of new electoral divisions alone cannot explain the restructuring of the European political space. The formation of new social cleavages also depends on the emergence of corresponding ‘organisational networks’ (Bartolini, 2000: 26); hence the project’s focus on interest politics at the organisational (meso) level. Furthermore, a study of labour mobilisations regarding NEG makes sense methodologically only if European integration is considered as a process ‘among distinct units indeed but, at the same time, units belonging to one single system’ (Caramani, 2015: 283). The project therefore aims **to go beyond methodological nationalism**. Whereas our transnational, economic sector and issue-oriented approach is riskier than conventional designs based on easily accessible national statistics and surveys, I am convinced that our design also promises high gains.

### Subject areas

Whereas the questions about the structuring of the European political space and the politicisation of European integration and NEG are discussed by sociologists and political scientists, the specific questions about labour’s capacity to enforce class compromises and democratic interest intermediation fall into the domain of industrial relations and social policy. For ‘several decades now the study of labour issues has been a specialist field’ (Crouch, 2015: 2). In the English-speaking world, this discipline used to be called industrial relations, until many universities merged it with ‘human resources management’. In continental Europe, *la question sociale* was a domain of social policy, which developed independently from Anglo-Saxon industrial relations. And yet, NEG may be bringing industrial relations and social policy together once more. These disciplines not only offer complementary vantage points, but are also directly affected by these ongoing changes. The latter might bring them back to the big questions about capitalism and democracy that led to the creation of the social sciences in the first place. Paradoxically, the closer alignment to management enabled industrial relations scholars to capture the governance by ‘coercive comparisons’ (Marginson and Sisson, 2004: 11) long before scholars in other disciplines theorised ‘governance by numbers’ (Supiot, 2015). ‘The increasing attention paid to “governance” may appear as reinventing the wheel. Industrial relations have always been characterised by interactions between public and private actors’ (Leonard et al., 2007: 6). Industrial relations also suggests that NEG’s governance by numbers will hardly lead to an end of social contestation. Multinational firms try to benefit from international competition by involving workers and unions from different sites in ‘whipsawing’ games (Greer and Hauptmeier, 2008: 77). And yet, Anner et al.’s study on the industrial determinants of transnational union solidarity also shows that ‘competition can frustrate cooperation, but it also motivates it’ (2006: 24).

### Restructuring the European political space

The labour movements triggered by the industrial revolution led to the formation of a European electorate and party systems along class cleavages, as shown by Caramani’s (2015) study of 150 years of voter alignment in 30 European states. But if one narrows the temporal focus of the analysis to ‘the age of globalisation’ then a new cleavage appears: namely, the cleavage between ‘winners’ and ‘losers’ of denationalisation processes (Kriesi et al., 2008). This cleavage has also been discussed as a conflict between cosmopolitan Europe-builders and Eurosceptic nationalists (Beck, 2002, 2013: 26 ff).

European labour parties and trade unions are indeed facing an increasingly Eurosceptic working class. In addition, the re-framing of socioeconomic conflicts in nationalistic terms by political and socioeconomic elites has been an important feature of labour politics since its inception. However, an analysis of the restructuring of the European political space cannot simply rely on quantitative data on voter attitudes. Equally important are organisational mobilisations and the political structures of opportunities in which these mobilisations are taking place (Tarrow, 1994). The processes that shape the European political space are *social* processes (Bartolini, 2005; Jabko, 2006; Saurugger, 2016). Individual attitudes become a social force only if they are mobilised. This depends on organisational networks located in the forecourt of party politics. This explains our interest in European unions, which still play a key role in the ‘organisational dimension’ of cleavage structuring (Allern and Bale, 2017; Bartolini, 2000).

Della Porta and Caiani (2009) avoid being captured by the politically charged conceptualisation of European protest movements along a unidimensional nationalism–cosmopolitanism axis. By analysing the frames used by particular European protest movements, they were able to highlight the fundamental differences between ‘critical Europeanists’ – who were for example active in the campaign against the so-called Bolkestein directive – and ‘populist Euroscepticism on which research has focussed in the past’ (Della Porta and Caiani, 2009: 135). However, although the distinction between progressive discourses of ‘critical Europeanists’ and regressive Eurosceptics worked well in social movements studies, the classification of protests based on discourses is problematic. I have therefore classified different European actor strategies leading to alternative EU-polity developments starting from actors’ activities rather than from their discourses (Erne, 2008: 21).

No European union is against a social and democratic Europe. And yet, there is a long list of cases in which organised labour mobilised along national cross-class rather than along transnational class lines. Our focus on meso-level organisational practices, instead of on programmatic statements and individual attitudes, therefore promises high gains. Similar conclusions emerge from our review of the politicisation literature.

### Politicising European governance

Political theorist Colin Hay conceptualised politicisation as a process that brings a subject into the realm of public deliberation and political choice (2007). Within European integration studies, politicisation is usually conceptualised as a process that can be empirically observed by studying (a) the growing salience of EU governance, involving (b) a polarisation of opinion and (c) an expansion of actors involved in EU governance (De Wilde et al., 2016: 4). If one compares the two conceptualisations however, two inconsistencies become apparent.

*Pace* De Wilde et al. (2016), the ‘salience of EU issues’ – for example in national media debates – is not necessarily a good indicator for European politicisation processes. If one follows Hay’s conceptualisation of the political as a realm of public choice, ‘not every mention of the EU should count as politicization’ (Zürn, 2016: 167).

*Pace* Hay (2007), the location of the entire ‘governmental sphere’ in the political realm is problematic, because it assumes that all governmental action is automatically located in the political realm. Governmental action has been increasingly delegated to ‘apolitical’ regulatory agencies, who conduct their actions as if they belong to the ‘non-political’ realm of necessity. This may or may not be a legitimate claim. But if there were no ‘private and governmental’ sphere, it would hardly make sense to talk about a politicisation of (EU) governance. Likewise, not all aspects of the individual and collective non-governmental spheres fall into the political ‘realm of contingency and deliberation’ either.

For this reason, I map the political realm in an alternative way. Although the claim of technocratic European governance institutions to be apolitical ‘often masks ideological choices’ (Weiler et al., 1995: 33), it is appropriate to locate their activities in the apolitical ‘realm of necessity’ (Hay, 2007). This revised conceptualisation maintains Hay’s conceptualisation of the political as the realm of public choice. In contrast to Hay (2007: 80) however, it puts the individual private sphere rather than the governmental sphere at the centre. This allows us to distinguish two types of governmental spheres at opposite ends of our map, namely, the public democratic and the private technocratic one. A policy issue remains ‘private and governmental’ as long as technocratic ‘regulatory governance’ is not challenged by social mobilisations for alternative public choices (Erne, 2008: 15). Hence, politicisation does not simply mean making technocratic governance subject to procedures of public scrutiny. Formal democratic procedures do not necessarily guarantee the availability of alternative public choices (Crouch, 2004; Mair, 2013).

Thus, we should know more about politicisation below the macro level of public debates as presented in mass media. We also need to know more about the role of interest groups and civil society organisations in the process of politicisation. This should not only open avenues for thicker descriptions of patterns of politicisation (Geertz, 1973), but also help to elucidate the consequences of politicisation in terms of equality and democracy (Zürn, 2016: 178).

## Research design

The more labour movements (old and new) politicise NEG in a transnational context, the more this will lead to restructuring the European political space along transnational class lines. In contrast, the more they politicise NEG in nationalist counter-mobilisations, the more this will lead to a fracturing of the European political space along national lines. Given the strong bias of NEG’s ‘corporate governance type’ structure in favour of intra-European competition, labour can also contribute to the fragmentation of the EU along national lines, through competitive adjustments or ‘beggar-thy-neighbour’ labour policies (Martin and Ross, 2004). Table 1 outlines the corresponding actor strategies and indicates observable activities, which allow their operationalisation in empirical research.

**Table 1.** Actor strategies leading to different structures of the European political space.

		*Observable actor activities leading to a restructuring of the political space:*	
*Action framework*		*along transnational lines*	*along national lines*
Politicising NEG (EU level)	EU-level contentious action Euro-strikes and demonstrations, European Citizens’ Initiatives	Yes	No
Depoliticising NEG (EU and/or national level)	No contentious action Support for NEG and competitive adjustments of labour policies	No	Yes
Politicising NEG (national level)	National contentious action Nationalist counter-mobilisations	No	Yes

Source: Adapted from Erne (2015: 305 and 2008: 25).

### Questioning methodological nationalism

So far, most studies on the popular responses to the Euro crisis and the new EU’s economic governance regime have relied on comparisons of different national cases (Bieler and Erne, 2015; Dufresne and Pernot, 2013; Hoffmann, 2015; Kriesi and Pappas, 2015; Stan et al., 2015; Vogiatzoglou, 2015). This is not surprising, given the dominance of methodological nationalism in the field, which mirrors approaches in terms of varieties of capitalism (Hall and Soskice, 2001), unionism (Crouch, 1993; Frege and Kelly, 2004; Hyman, 2001) and welfare regimes (Esping-Andersen, 1990). The design of Gumbrell-McCormick and Hyman’s (2013) study is representative of the field. However, designs that are exclusively based on national variables are unable to capture the restructuring of the economy and society along transnational supply and value chains (Dicken, 2011) or along transnational ‘care chains’ (Stan and Erne, 2014) and labour control regimes (Anner, 2015). Accordingly, the workings of NEG – and the union and social movement activities that are being triggered by it – cannot be adequately captured by national statistics and datasets either. Social mobilisations that politicise NEG have to be studied (a) at the meso level of interest politics and (b) within *and* across national boundaries. Hence, I am making the case for the disaggregation of the units under study. This contextualised approach to the study of labour politics will enable us to capture and compare social dynamics that often fall under the radar of macro-level comparisons (Locke and Thelen, 1995).

Concretely, I am proposing a research design that is no longer based on the comparison of national units. Instead, I am proposing an alternative design that compares the workings of NEG and labour movements in different areas of labour politics and in different economic sectors. This includes investigations at EU level, but also enquiries in selected countries as well as parallel case studies. In contrast to the approaches in terms of varieties of capitalism, unionism and welfare states however, the selection of locations for empirical analysis will not be informed by different ‘types’ of national regimes. Instead, sub-EU locations will be selected in order to capture both central and peripheral locations in the uneven European political and economic space.

### Case selection

We will examine the workings of NEG in two areas of labour politics **(wage policy and the provision of public services)** and in three sectors **(healthcare, transport, and water services)**. These areas and sectors are all directly affected by NEG, albeit in different ways. Wage policy is affected by interventions targeting collective wage bargaining and labour law. The provision of public services is directly affected by NEG’s interventions in national budgets and the social field (Clauwaert, 2014). This case selection enables us to capture not only vocal reactions, e.g. contentious action. It also allows us to capture cooperation with NEG, e.g. union cooperation in the implementation of requested competitive wage adjustments. This allows us to observe actor activities in relation to both politicisation *and* depoliticisation (see Table 1).

Wage policy and the provision of public services also differ in relation to the social actors involved. Whereas unions tend to prioritise wages, social movements are more concerned about citizens’ access to public services. At times the two concerns converge, however, as in the case of the ‘*right2water’* European Citizens’ Initiative (ECI) of the European Federation of Public Sector Unions (Bieler, 2015). At times, they do so to a lesser extent, as in the case of the ‘fair transport’ ECI of the European Transport Workers Federation. This allows comparisons across areas of labour politics that are usually studied by distinct disciplines. Finally, the healthcare, transport and water services sectors are not only relevant because they are all directly affected by NEG interventions. They are also affected by horizontal federalising dynamics, caused for example by the free movement of workers, services, and patients. This allows us to compare and contrast NEG with horizontal EU integration processes.

### Methods

Our empirical work will be based on ‘multi-sited’ (Marcus, 1995) fieldwork on NEG and wage bargaining and the provision of services of general interest in different sectorial, national and transnational locations. This will involve (1) expert interviews with national and EU-level officials from unions, employers’ associations and social movements, as well as civil servants and politicians, (2) participant observations of public activities of the organisations under study, (3) and an analysis of their documents. Given past experience (Erne, 2008), I am very confident that I will get access to all relevant actors in the field. I am also familiar with the sectorial, social dynamics in the field, not only due to my research experience in European labour relations (Erne, 2008, 2015; Stan and Erne, 2014, 2016) and direct and transnational democracy (Erne et al., 1995), but also because of my past experiences as a trade union and new social movement activist. However, precisely for this reason, I am aware that the analysis of transnational social dynamics that are at work in a specific sector requires deep knowledge and extensive language skills. I shall therefore advertise a position for a Senior Research Fellow (50%) and two post-doctoral positions (100%) for researchers with particular language and research experience in healthcare, transport and water services. Furthermore, we will also offer three full PhD fellowships in conjunction with the structured joint PhD programme offered by the UCD Michael Smurfit Graduate School of Business and the UCD Graduate School of the College of Social Sciences and Law.

### Schedule

In **year 1,** we will map NEG’s interventions in the area of wage bargaining and the provision of public services; and the politicisation and depoliticisation paths they entail. This includes an examination of NEG’s country-specific recommendations and corrective action plans and an analysis of the related submissions of interest groups to the Commission. We will also conduct a first set of preliminary, case studies (involving each one month of fieldwork in the health, transport and water services sectors at EU level) in relation to actor’s activities regarding NEG, wage bargaining and the provision of public services. We will also draw a new typology of European countries, according to their central or peripheral location in the European economic and political space. This will allow us to go beyond the traditional, institutionalist approaches in comparative labour politics in terms of varieties of capitalisms, unionism and welfare states. This typology will then be used for the selection of appropriate fieldwork locations in years 2 and 3.

The **second and third year** of the project will be dedicated to the project’s third objective: to analyse the responses of established unions and new labour movements to NEG as well as their feedback effects on NEG in the two areas of labour politics under investigation in three sectors (healthcare, transport and water services). To this effect, we will conduct extended fieldwork at EU level and in selected national locations. At the beginning of year 2, we will also organise our first international peer-review workshop, with leading European specialists in the field of qualitative and comparative methodology, namely, “multi-sited” fieldwork in institutional and labour movement contexts in transnational governance, production and reproduction chains (Marcus, 1995). This occasion will allow us to present and discuss the first journal articles that will be based on the conceptual and methodological work as well as the findings of our pilot sectorial case studies and NEG mapping exercises conducted in year 1. At the end of year 3, we will organise another peer-review workshop to discuss the findings of our fieldwork in the healthcare, transport and water services with leading national and European experts in the field.

The **fourth year** will be dedicated to the comparative analysis of our empirical findings across sectors and subject areas and the writing of two special journal issues or edited books that will result from the project’s fieldwork in years 2 and 3. The **final year** will be dedicated to the finalisation and discussion of our monograph, which aims to attain the project’s fourth and final objective: to develop a new scientific paradigm capable of accounting for the interplay between EU economic governance, labour politics and EU democracy. We plan to discuss the draft book manuscript at our last peer-review workshop, with world-leading scholars of old and new labour movements, (de-)democratisation processes, the structuring and (de-)politicisation of the European political and socioeconomic space.

## Conclusion

The big questions addressed in this project are relevant not only for the future of democracy and social justice, but also for the predominately institutionalist approaches in my field. I believe that the growing horizontal and vertical integration of Europe, and the counter-movements that these processes are triggering, are calling for a paradigm shift. I am therefore planning to publish a monograph, in which I will not only present the overall findings our research, but also demonstrate that there are promising new methodological paths that go beyond the methodological nationalism in my field.

Roland ErneUCD School of Business and Geary Institute for Public Policy, Belfield, Dublin, Ireland Email: Roland.Erne@ucd.ie
